# Incorporating Fatty Acids Enhanced the Performance of Konjac Glucomannan/Chitosan/Zein Film

**DOI:** 10.3390/foods14091563

**Published:** 2025-04-29

**Authors:** Xiumei Wang, Yibin Chen, Xiaoxu Zhao, Jie Pang

**Affiliations:** 1 College of Environmental and Biological Engineering, Putian University, Putian 351100, China; wxm890626@163.com (X.W.); chenyb0866@163.com (Y.C.); 2 Fujian Provincial Key Laboratory of Ecology-Toxicological Effects & Control for Emerging Contaminants, Putian University, Putian 351100, China; 3 Key Laboratory of Ecological Environment and Information Atlas of Fujian Provincial University, Putian University, Putian 351100, China; 4 College of Food Science, Fujian Agriculture and Forestry University, Fuzhou 350002, China

**Keywords:** konjac glucomannan, chitosan, Zein, saturated fatty acids, biodegradable packaging films, properties

## Abstract

The effects of stearic acid (SA) and lauric acid (LA) with different concentrations on the structure and physicochemical properties of konjac glucomannan (KGM)/chitosan (CTS)/Zein (KCZ) film were systematically investigated in this paper. The rheology results suggested that the apparent viscosity of the KCZ film solution was significantly enhanced after adding fatty acids (FAs), and all the film-forming solutions were typical non-Newtonian pseudoplastic fluids. Hydrogen bond interactions were formed among KGM, CTS, Zein, and FA molecules. KCZ-FA films had higher crystallinities than KCZ film, and their crystallinities increased with the increase in FA concentrations. Microstructure indicated that adding FAs significantly affected the surface morphologies and roughness of KCZ film. KCZ-LA films exhibited much rougher surfaces than KCZ-SA films when FA concentrations were the same. Moreover, the incorporation of FAs significantly (*p* < 0.05) decreased the transmittance of KCZ film. KCZ-FA films exhibited higher hydrophobicities and water vapor barrier properties than KCZ film due to their significantly (*p* < 0.05) higher water contact angle, lower water solubility, water content, and vapor permeability values. The thermal stabilities, color attributes, and mechanical properties of KCZ film were also improved after adding appropriate concentrations of FA. Therefore, KCZ-FA films with excellent performances are promising food packaging materials in the future.

## 1. Introduction

In recent decades, environmentally friendly biodegradable films have attracted much attention due to the serious food safety and white environment pollution problems caused by the non-degradable petroleum-based packaging films [1,2,3,4]. Moreover, it is found that those biodegradable films made of various natural polymers such as proteins, polysaccharides, and lipids can extend food shelf life and improve food safety by inhibiting microbial reproduction, delaying oxidation, preventing food water loss, etc. [5,6,7]. Therefore, the development of novel biodegradable films with better properties has become a hot topic in the food packaging field.

Konjac glucomannan (KGM), composed of β-1,4-linkage-linked α-mannose and α-glucose, is a water-soluble, neutral, renewable polysaccharide with high molecular weight and has been widely used in the food packaging field due to its excellent film-forming ability, good biodegradability, and biocompatibility [8,9,10]. However, pure KGM film has poor mechanical strength, water resistance, and thermal stability [11,12,13]. Among various methods to overcome these issues, blending KGM with other biodegradable components has been confirmed to be an effective way to improve the performances [14,15]. It was discovered that adding chitosan (CTS) could enhance the mechanical properties of pure KGM film [16,17,18]. Nevertheless, the water resistance and thermal stability of KGM/CTS film were still poor due to the strong hydrophilicities of natural KGM and CTS molecules [19,20]. Considerable studies have shown that the properties of polysaccharide films could be enhanced after the incorporation of proteins [21,22,23]. Zein, a natural plant protein, has been blended with various polysaccharides to produce the composite films with better barrier properties and thermal stability due to its low gas permeability and high non-polar amino acid level [24,25,26]. However, the water vapor barrier performance of the composite films formed by protein and polysaccharide still cannot meet the current requirements of food packaging films and needs to be further enhanced [27,28]. Therefore, it is urgent and promising to prepare protein-polysaccharide composite films with better moisture barrier properties by introducing more hydrophobic constituents.

Recent research has shown that fatty acids (FAs) such as palmitic acid, stearic acid (SA), and lauric acid (LA) exhibit very high hydrophobicity and have been extensively applied for the preparation of food packaging films with better moisture barrier properties [29,30,31]. Thus, blending protein-polysaccharide composite films with fatty acids is an approach to achieve enhanced water vapor barrier properties [32]. Moreover, to the best of our knowledge, KGM/CTS/Zein/fatty acid (KCZ-FA) films have never been reported in the current literature. Considering that excessive temperatures may cause protein denaturation and unsaturated fatty acids are prone to oxidation, two common saturated fatty acids with mild melting points were selected in this paper, namely, SA and LA [29]. Therefore, this paper was to evaluate the effects of SA and LA on the structure and properties of KCZ film. Firstly, KCZ films with different concentrations of SA and LA were prepared through a solution casting method. Then, the apparent viscosities of all the film-forming solutions were measured. Subsequently, KCZ-FA films were characterized by Fourier transform infrared spectroscopy (FTIR), differential scanning calorimetry (DSC), thermogravimetric analyzer (TGA), X-ray diffractometer (XRD), scanning electron microscopy (SEM), and atomic force microscope (AFM). The mechanical properties, opacity, color, water content (WC), solubility (WS), vapor permeability (WVP), and contact angle (WCA) of KCZ-FA films were also studied. Finally, the potential formation mechanism of KCZ-FA films was discussed.

## 2. Materials and Methods

### 2.1. Materials

Lauric acid (98%) was obtained from Shanghai Maclean’s Biochemical Technology Co., Ltd. (Shanghai, China). Zein and acetic acid were supplied by Adamas-beta Co., Ltd. (Shanghai, China). Konjac glucomannan (purity ≥ 95%), chitosan (deacetylation degree ≥ 95%), stearic acid (98%), and anhydrous calcium chloride were purchased from Shanghai Aladdin Biochemical Technology Co., Ltd. (Shanghai, China). Anhydrous ethanol and glycerol were sourced from China National Pharmaceutical Group Chemical Reagent Co., Ltd. (Shanghai, China).

### 2.2. Film Preparation

Pure KGM film solution was prepared under the conditions of 0.75 g KGM, 0.05 g glycerol, and 100 mL of distilled water with mechanical stirring at 70 °C for 1 h. Pure CTS film solution was prepared by mixing 2 g CTS and 0.05 g glycerol in 100 mL of 1% acetic acid with mechanical stirring at 70 °C for 1 h. Pure Zein film solution was prepared by mixing 2 g of Zein and 1 g of glycerol in 100 mL of 80% ethanol with mechanical stirring at 35 °C for 45 min.

The KC film solution was obtained by mixing pure KGM and CTS film solutions with the volume ratio of 5:1 at 70 °C for 30 min. The KZ film solution was obtained by mixing pure KGM and Zein film solutions with the volume ratio of 10:1 at 70 °C for 20 min. The KCZ film solution was obtained by mixing pure KGM, CTS, and Zein film solutions with the volume ratio of 10:2:1 at 70 °C for 30 min.

**Figure 1:**
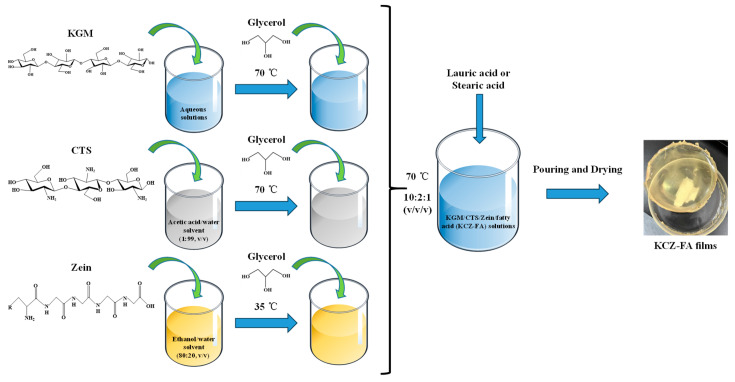
The preparation process of KGM/CTS/Zein/fatty acid (KCZ-FA) films.

For KCZ-FA film solutions (Figure 1), LA (0.1, 0.2, 0.3, 0.4, and 0.5% pure KGM film solution) or SA (0.02, 0.04, 0.06, 0.08, and 0.10% pure KGM film solution) was added to KCZ film solution with stirring at 70 °C for 30 min, respectively. After that, glass Petri dishes containing these single, binary, ternary, and quaternary film solutions were dried in an oven at 60 °C for 6, 5, 5, and 4 h, respectively. Finally, all the dried films were peeled from glass Petri dishes and stored in a constant temperature and humidity box (relative humidity of 50% and temperature of 25 °C) before use. The films were sequentially designated as KGM, CTS, Zein, KC, KZ, KCZ, KCZ-LA1, KCZ-LA2, KCZ-LA3, KCZ-LA4, KCZ-LA5, KCZ-SA2, KCZ-SA4, KCZ-SA6, KCZ-SA8, and KCZ-SA10.

### 2.3. Rheological Properties of Various Film Solutions

The apparent viscosities of various film solutions were measured by a Hack Rheometer (Viscotester iQ, Hack Corporation, Kurtscheid, Germany) equipped with FL 22 4B/SS-01160184 rotor at 25 °C. The rotor speed was 45 r/s and the measurement time was 30 s. During this period, a total of 15 data points were taken.

### 2.4. FTIR Analysis of Various Films

Before analysis, various films were cut into 2 mm × 2 mm, small, square pieces. Then, FTIR spectra of various films were acquired using an FTIR spectrometer (TENOSOR27, Bruker Corporation, North Billerica, MA, USA) in the wavelength range of 400–4000 cm^−1^ at a resolution of 4 cm^−1^, for a total of 32 scans.

### 2.5. XRD Analysis of Various Films

Before analysis, various films were cut into circular disks with a diameter of 2 cm. Subsequently, the crystalline structures of various films were characterized using an X-ray diffractometer (XRD-6100, Shimadzu Corporation, Kyoto, Japan) with a Cu-Kα radiation source at 40 kV and 30 mA. The scanning angular range was 5–40° (2θ) and the scanning speed was 4°/min.

### 2.6. Thermal Stabilities Analysis of Various Films

The thermal stabilities of various films were investigated by thermogravimetric analyzer (TGA) and differential scanning calorimetry (DSC). The DSC analysis of various films was performed on a NETZSCH DSC 214 (Netzsch Co, Selb, Bavaria, Germany) according to the previously described method [12,32] with some modifications. Before analysis, various films were cut into small pieces. Subsequently, these small pieces were placed in hermetically sealed aluminum pans. After that, all the films were heated from 40 to 200 °C at a heating rate of 10 °C/min and maintained at 200 °C for 5 min under a nitrogen (N_2_) atmosphere with a flow rate of 50 mL/min.

The TGA analysis of various films was carried out by a thermogravimetric analyzer (SDT650, Waters Corporation, Milford, MA, USA) under a N_2_ atmosphere with a flow rate of 50 mL/min. Before the measurement, approximately 10 mg of various films was sealed in the ceramic crucibles. Subsequently, various films in the sample cells were heated from room temperature to 600 °C at a rate of 10 °C/min.

### 2.7. Microstructure Analysis of Various Films

Before the observation, all the films were cut into a size of 2 cm × 1 cm strips and attached to the sample stage with conductive glue. After that, the obtained samples were sprayed with gold under vacuum. Finally, the surface morphologies of various films were observed and recorded by a SEM (SU-8010, Hitachi Ltd., Tokyo, Japan) at an accelerating voltage of 10 kV and 1000-fold magnification.

Before the measurement, various films were cut into a square (5 mm × 5 mm) and then fixed on the sample table. Subsequently, the surface roughness of various films was observed by atomic force microscope (AFM) (Bruker Dimension ICON, Bruker, Germany) in tapping mode at room temperature. NanoScope software version 5.31 was used for AFM image processing, and the roughness parameters of average roughness (Ra) and root-mean-square roughness (Rq) of various films were obtained.

### 2.8. Mechanical Properties of Various Films

The elongation at break (EB) and tensile strength (TS) of various films were determined using a CT3-4500 Texture Analyzer (Ametek Corporation, Berwyn, PA, USA). Before analysis, various films were cut into long strips (70 mm × 20 mm) and a spiral micrometer (DL9325, Deli Group Co., Ltd., Ningbo, China) was employed to measure the thicknesses of these strips. Then, these strips were fixed on the TA-DGA probe for tensile testing. The initial grip length, a trigger point load, and the testing speed were 50 mm, 0.1 N, and 0.5 mm/s, respectively. TS (MPa) and EB (%) values were calculated using Equations (1) and (2), respectively.

(1) $$TS=\frac{F}{W\times d}$$

(2) $$EB=\frac{L-L_{0}}{L_{0}}\times 100$$

where *F* (N) represents the maximum force, *W* (mm) and *d* (mm) mean the width and thickness of various films, *L* (mm) and *L*_0_ (mm) reflect the final and original lengths of various films, respectively.

### 2.9. Water Content (WC), Solubility (WS), Vapor Permeability (WVP) and Contact Angle (WCA) of Various Films

The WC of various films was measured as follows: before the measurement, various films were cut into a square (20 mm × 20 mm). Then, the square was weighed and placed in an oven at 105 °C for drying. After drying for 24 h, the square was taken out and weighed again. The WC of various films was calculated using Equation (3).

(3) $$WC(\%)=\frac{m_{1}-m_{2}}{m_{1}}\times 100$$

where *m*_1_ (g) and *m*_2_ (g) were the mass of various films before and after drying, respectively.

The WS of various films was measured as follows: various dried films (2 cm × 2 cm) were first immersed in 30 mL of distilled water at room temperature for 24 h. Subsequently, various insoluble films after pouring out the leaching solution were dried again at 105 °C until the mass reached a constant value. The WS of various films was calculated using Equation (4).

(4) $$WS\left(\%\right)=\frac{m_{3}-m_{4}}{m_{3}}\times 100$$

where *m*_3_ (g) was the mass of various films before the immersion, *m*_4_ (g) was the mass of various films after immersing and drying.

The WVP of various films was measured according to the methods of Zhou et al. (2022) [33] and Chang et al. (2021) [34] with some modifications. The glass cups containing 3 g of anhydrous calcium chloride were sealed with various films. Then, the glass cups were weighed and placed at a temperature of 25 °C with a relative humidity of 90%. During this process, the mass of the glass cups was weighed regularly every 1 h until the increase in mass before and after the two tests was less than 5%. The WVP (g∙m^−1^·s^−1^·Pa^−1^) was calculated using Equation (5).

(5) $$WVP=\frac{\Delta m\times L}{S\times t\times \Delta P}$$

where Δ*m* (g) means the increase in mass of the glass cups, Δ*P* (Pa) means the water vapor pressure difference between the two sides of various films, *L* (m) means the thickness of various films; *S* (m^2^) means the effective area of various films, *t* (s) means the interval time between the measurements.

The WCA of various films was determined using the sessile drop method by a fully automatic contact angle measuring instrument (SL250, KINO Scientifc Instrument Inc., Boston, MA, USA) at room temperature. Before the measurement, various films were cut into a rectangle (10 mm × 20 mm) and attached to the sample stage with adhesive tape. Then, approximately 10 μL of distilled water per drop was placed on the surfaces of various films using a precise syringe. Subsequently, photos were obtained after the water drops were left stable for around 30 s. Finally, The WCA values of various films were calculated using Young-Laplace equation.

### 2.10. Color Analysis of Various Films

The color of various films was analyzed by a colorimeter (10QC, Shenzhen San’enshi Technology Co., Ltd., Shenzhen, China). Before analysis, the colorimeter was calibrated by a standard white board ($L_{0}^{*}$ = 90.84, $a_{0}^{*}$ = 1.26, $b_{0}^{*}$ = −0.80). Subsequently, L* (light or dark), a* (red or green) and b* (yellow or blue) values of various films were acquired using the standard white board as the background. The total color difference (ΔE*) of various films was calculated using Equation (6).

(6) $$\Delta E^{*}=\sqrt{{\left(L^{*}-L_{0}^{*}\right)}^{2}+{\left(a^{*}{-a}_{0}^{*}\right)}^{2}+{\left(b^{*}-b_{0}^{*}\right)}^{2}}$$

### 2.11. Opacity Analysis of Various Films

The opacity analysis of various films was carried out using a dual beam ultraviolet visible spectrophotometer (TU-1900, Beijing Puxi General Instrument Co., Ltd., Beijing, China). Before analysis, various films were cropped to a rectangle (10 mm × 40 mm) and then attached to one side of the cuvette. Subsequently, the measurements were conducted at a wavelength of 600 nm using an empty cuvette as the blank control. Finally, the opacity of various films was calculated using Equation (7).

(7) $$Opacity=\frac{A_{600}}{d}$$

where *A*_600_ means the absorbance of various films at 600 nm and *d* (mm) represents the thickness of various films.

### 2.12. Statistical Analysis

All the experiments were repeated in triplicate. The results were expressed as the means ± standard deviations (SD). The statistical analysis of all the experimental data were conducted using Origin 8.0 software and SPSS 26 software. Significant differences (*p* < 0.05) among the means were determined through the analysis of variance, Duncan’s multiple range tests, and Least significant differences (LSD) multiple comparison tests.

## 3. Results and Discussions

### 3.1. Rheological Properties of Various Film Solutions

**Figure 2:**
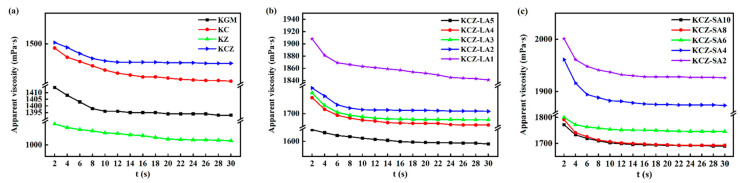
Rheological properties of various film solutions. (**a**) KGM, KC, KZ, KCZ film solutions; (**b**) KCZ-LA film solutions; (**c**) KCZ-SA film solutions.

Figure 2 shows the effects of FAs on the rheological properties of KCZ film solution. As seen from Figure 2, incorporating Zein decreased the viscosity of pure KGM film solution. However, adding CTS enhanced the viscosities of pure KGM and KZ film solutions, indicating that the network structures of KC and KCZ film solutions were strengthened. This is probably because the molecular chain segments of CTS intertwined with KGM and/or Zein to form an ordered structure during the stirring process, resulting in good gel properties. It was also observed that KCZ-FA film solutions showed higher viscosities than KCZ film solutions, indicating that FA was embedded in the network structure of KGM, CTS, and Zein. Nevertheless, all the film solutions presented a gradually decreasing trend in the viscosity as the shear time increased, which showed a shear-thinning behavior. The results suggested that all the film solutions were typical pseudoplastic fluids. This may be probably because polymer chains were unraveled and rearranged under shear force [14,35]. In addition, the viscosities of KCZ-FA film solutions decreased with the increase in FA concentrations. This may be probably because of a reduction in solvent quality and the destruction of hydrogen bonds among KGM, CTS, and Zein caused by the presence of FAs, resulting in the shrinkage or the conformation change in polymer chains. Moreover, it was found that the KCZ-SA film solution had lower viscosity than the KCZ-LA film solution when SA and LA concentrations were both 0.1%. Therefore, the viscosity of the KCZ film solution was significantly affected by FA types and concentrations.

### 3.2. FTIR Analysis of Various Films

**Figure 3:**
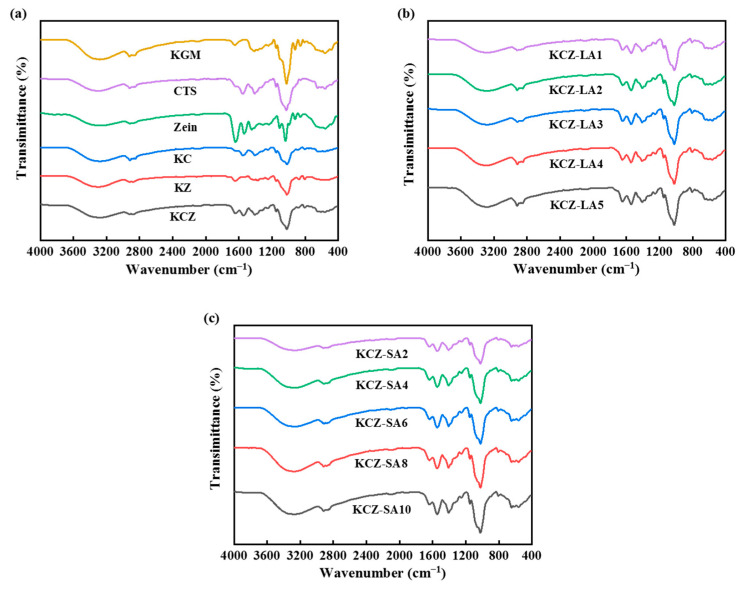
FTIR spectra of various films. (**a**) KGM, CTS, Zein, KC, KZ, and KCZ films; (**b**) KCZ-LA films; (**c**) KCZ-SA films.

The FTIR spectra of various films are shown in Figure 3. For pure KGM film, the absorption peaks at 3280, 1734, and 1027 cm^−1^ were attributed to the stretching vibration of the hydroxyl group, C=O in the acetyl group, and C–O–C groups, respectively [36]. The peak at 1645 cm^−1^ was related to intramolecular hydrogen bonds [37]. The peaks at 2921 and 2860 cm^−1^ were ascribed to C–H stretching vibration of the methyl in KGM [38]. The characteristic absorption peaks at 858 and 808 cm^−1^ represented the stretching vibration of the mannose units in KGM [39]. For pure CTS film, the absorption peaks at 3288 and 1650 cm^−1^ represented the stretching vibration of –OH and C=O bonds, respectively. The symmetrical stretching vibration of CH_2_ bonded to –OH occurred at 2926 and 2867 cm^−1^. The absorption peaks at 1153 and 1030 cm^−1^ represented bridge oxygen structure C–O–C stretching vibration. The N–H stretching vibration peaks appeared at 1553, 1420 and 1327 cm^−1^ [4]. For pure Zein film, the absorption peaks at 3292, 1650, 1539, 1450, and 1030 cm^−1^ were correlated to the vibration of N–H stretching, C=O stretching in the amide I, N–H bending in the amide II, –CH_2_ bending, and C–N stretching in the amide III, respectively. The absorption peaks at 2926 and 2867 cm^−1^ represented the vibration of C–H stretching in the aliphatic groups [10].

It was also seen from Figure 3 that there were no new absorption peaks appearing in the spectra of KC, KZ, KCZ, and KCZ-FA films when compared to the spectra of pure CTS, Zein, and KGM films, indicating that no chemical reactions happened during the preparation processes of these composite films. However, adding FAs to KCZ film resulted in the shift in the –OH stretching vibration peak to higher wavenumber and broadened C–H and C=O peaks, which may be due to the formation of hydrogen bonds among the four substances. Moreover, the intensities of C–H and C–O stretching vibration peaks in the KCZ-FA films gradually increased with the increase in FA concentrations. It was also discovered that KCZ-FA films had similar positions of IR absorption peaks to KCZ film. These results suggested that the structure of KCZ film was not significantly affected by FAs, but hydrogen bond interactions among the four substances may lead to better mechanical and physicochemical properties.

### 3.3. XRD Analysis of Various Films

**Figure 4:**
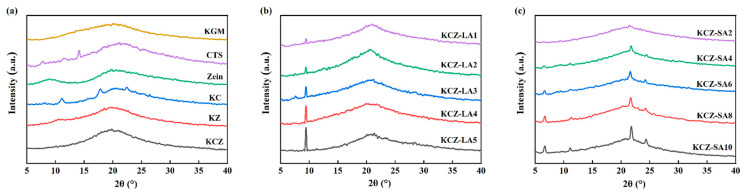
XRD patterns of various films. (**a**) KGM, CTS, Zein, KC, KZ, and KCZ films; (**b**) KCZ-LA films; (**c**) KCZ-SA films.

Figure 4 shows the XRD patterns of various films. As seen from Figure 4, pure KGM film presented a broad diffraction peak at 2θ = 20°, suggesting that KGM was an amorphous material, which was in agreement with some previous reports [12,40]. Two broad diffraction peaks at 2θ = 8.8° and 20.1° were observed in the XRD pattern of pure Zein film, which was consistent with the previous results [29,41]. This may be probably because the crystallinity of Zein was weak due to the disordered protein structure. Thus, no new diffraction peaks were observed in the XRD pattern of KZ film when compared to pure KGM and Zein films, but the diffraction peak of pure Zein film at 2θ = 8.8° shifted to the right in the XRD pattern of KZ film. Moreover, it was found that CTS film displayed narrow diffraction peaks at 2θ = 7.8°, 11.5°, and 14.1° and a broad diffraction peak at 2θ = 21.3°, respectively. These narrow diffraction peaks of pure CTS film shifted to the right in the XRD pattern of KC film due to the intermolecular interactions between KGM and CTS [19,42,43]. However, only one broad diffraction peak at 2θ = 20° was discovered in the XRD pattern of KCZ film. This may be due to the formation of strong hydrogen bonds between Zein and CTS, which destroyed the original crystalline domains of Zein and CTS [23,44]. The crystalline structure of KCZ film was also affected after incorporating FAs. Compared with KCZ film, the diffraction peak at 2θ = 20° shifted to a higher diffraction angle and became narrow in the XRD patterns of KCZ-LA films, whereas this peak disappeared in the XRD patterns of KCZ-SA films. In addition, a new sharp diffraction peak at 2θ = 9.42° gradually emerged in the XRD patterns of KCZ-LA films, whereas four new sharp diffraction peaks appeared at 2θ = 6.6°, 11°, 21.7°, and 24.3° in the XRD patterns of KCZ-SA films, which corresponded to the characteristic diffraction peaks of LA and SA, respectively [32]. More importantly, it was found that there was no significant change in the crystalline peak positions of KCZ-FA films containing different FA concentrations, but the intensities of these new peaks increased as FA concentrations increased. This is probably because intermolecular hydrogen bonds among KGM, CTS, Zein, and FAs increased as FA concentrations increased. These results indicated that adding FAs to KCZ film was beneficial to enhance the degree of crystallization.

### 3.4. Thermal Stabilities of Various Films

**Figure 5:**
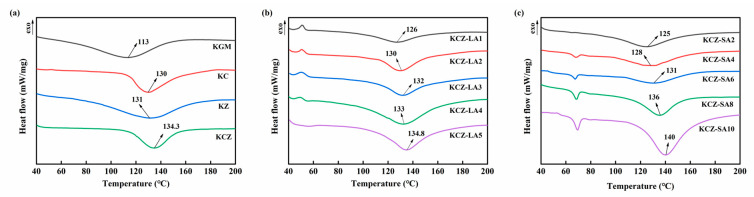
DSC curves of various films. (**a**) KGM, KC, KZ, and KCZ films; (**b**) KCZ-LA films; (**c**) KCZ-SA films.

The effects of FAs on the thermal stabilities of KCZ film were evaluated by means of DSC and TGA measurements. The results of DSC measurements are shown in Figure 5. The endothermic peak in the DSC curve represents the glass transition temperature (Tg) of the film. A single Tg represents good compatibility of the substances [45]. It was observed that the DSC curves of pure KGM, KC, KZ, and KCZ films showed a single Tg at 113, 130, 131, and 134.3 °C, respectively. The results indicated an improvement in the thermal stabilities of binary and ternary composite films. This may be due to the good compatibility among KGM, CTS, and zein molecules. In addition, the Tg of KCZ-FA films increased with the increase in FA concentrations. However, KCZ-LA films had lower Tg than KCZ film when LA concentrations were below 0.4%. Only KCZ-LA5 film was discovered to have a slightly higher Tg than KCZ film. Moreover, a single Tg in the DSC curves of KCZ-LA films indicated good compatibility of KGM, CTS, Zein, and LA. As for KCZ-SA films, there were two endothermic peaks in the DSC curves. The first endothermic peak was the melting temperature of SA at around 69 °C [32,46] and the second endothermic peak represented the Tg of KCZ-SA films. This is probably due to the heterogeneous distribution of SA with long carbon chains in the composite films [47]. It was also found that KCZ-SA films had lower Tg than KCZ film when SA concentrations were below 0.06%. After that, KCZ-SA films had higher Tg than KCZ films. This may be probably because of stronger intermolecular interactions in the composite film matrix with the increase in SA concentrations, resulting in higher thermal stabilities of KCZ-SA films when SA concentrations exceeded 0.06%. More importantly, it was discovered that KCZ-SA films had higher Tg than KCZ-LA films when SA and LA concentrations were both 0.1%. The above results indicated that adding appropriate concentrations of SA could better enhance the thermal stability of KCZ films.

**Figure 6:**
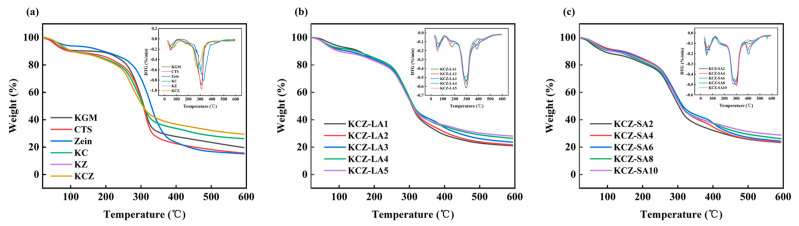
TGA and DTG curves of various films. (**a**) KGM, CTS, Zein, KC, KZ, and KCZ films; (**b**) KCZ-LA films; (**c**) KCZ-SA films.

Figure 6 shows the results of TGA measurements. The slight weight loss in the initial stage before 130 °C was due to the evaporation of non-bound water and/or solvent in the films. The second stage, around 130~210 °C in the films, was related to the evaporation of glycerol and bound water. The major weight loss around 210~420 °C was attributed to the thermal decomposition of KGM, CTS, Zein, and FAs. It was observed that compared with KCZ film, the decomposing stages of KCZ-FA films were postponed, indicating that their thermal stabilities had been improved in this stage. This may be probably due to the stronger interactions among KGM, CTS, Zein, and FAs. However, it was discovered that the initial thermal degradation temperatures of KCZ-FA films changed irregularly as FA concentrations increased. After that, the weight loss of various films was greatly slowed down from around 420 °C. In addition, it was worth to note that the weight loss of KCZ-FA films decreased with the increase in FA concentrations in the final stage. More importantly, it was found that the weight loss of KCZ-SA film was lower than that of KCZ-LA film when SA and LA concentrations were both 0.1%. The above results suggested that the thermal stability of KCZ film was affected by the types and concentrations of fatty acids.

### 3.5. Microstructure of Various Films

**Figure 7:**
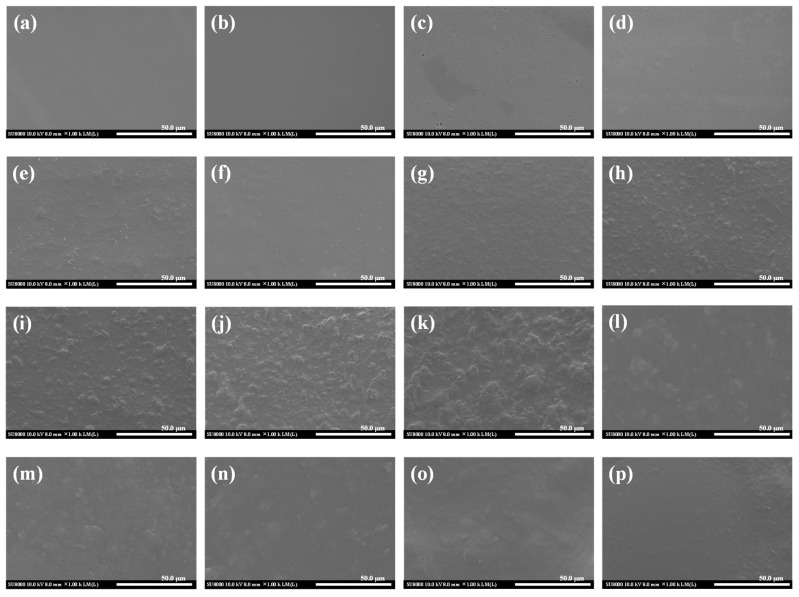
SEM images of various films. (**a**) Pure KGM film; (**b**) pure CTS film; (**c**) pure Zein film; (**d**) KC film; (**e**) KZ film; (**f**) KCZ film; (**g**) KCZ-LA1 film; (**h**) KCZ-LA2 film; (**i**) KCZ-LA3 film; (**j**) KCZ-LA4 film; (**k**) KCZ-LA5 film; (**l**) KCZ-SA2 film; (**m**) KCZ-SA4 film; (**n**) KCZ-SA6 film; (**o**) KCZ-SA8 film; (**p**) KCZ-SA10 film.

Figure 7 shows the effects of FAs on the surface morphologies of KCZ film. It was observed that pure KGM and CTS films had smooth and homogeneous surfaces, similarly to the previous reports [42,48]. The surface of KC film was also relatively smooth and compact, indicating that KGM and CTS had good compatibility [20,43,49]. In addition, it was found that the surface of pure Zein film was smooth with black tiny holes, which may be probably because of the encapsulated air microbubbles [14,45]. However, the surface of KZ film was a little rough and irregular. The reason was that the agglomerations of Zein and the intermolecular interactions between KGM and Zein appeared in the film matrix [50]. Compared with the KZ film, the surface of the KCZ film presented a relatively smooth structure with slight agglomerations, which may be due to the increasing number of hydrophilic groups on the surface. Nevertheless, incorporating FAs significantly changed the surface morphologies of KCZ film. The surface roughness and irregularity of KCZ-LA films increased with the increase in LA concentrations. When LA concentration was 0.5%, the surface of KCZ-LA film was the roughest and most irregular. This may be due to the agglomerations of the polymers in the film matrix. Although KCZ-SA films exhibited relatively rougher surfaces than KCZ films, their surface roughness and irregularity increased first and then decreased with the increase in SA concentrations. This may be mainly attributed to the hydrogen bond interactions and good compatibility among KGM, CTS, Zein and SA. It was also discovered that the KCZ-LA film had a more compact and rougher surface than the KCZ-SA film when SA and LA concentrations were both 0.1%. The above results indicated that the surface morphologies of KCZ film were significantly influenced by FA types and concentrations.

**Figure 8:**
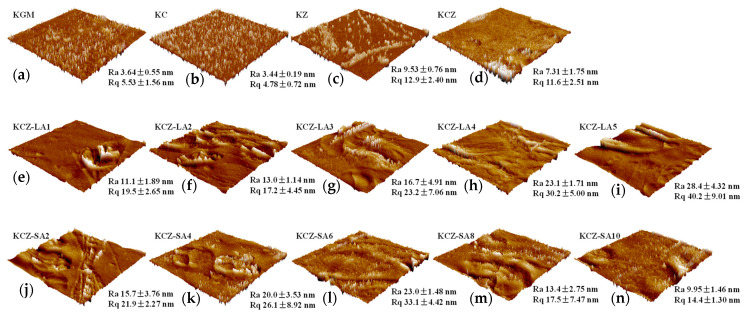
AFM images of various films. (**a**) Pure KGM film; (**b**) KC film; (**c**) KZ film; (**d**) KCZ film; (**e**) KCZ-LA1 film; (**f**) KCZ-LA2 film; (**g**) KCZ-LA3 film; (**h**) KCZ-LA4 film; (**i**) KCZ-LA5 film; (**j**) KCZ-SA2 film; (**k**) KCZ-SA4 film; (**l**) KCZ-SA6 film; (**m**) KCZ-SA8 film; (**n**) KCZ-SA10 film.

Figure 8 shows the effects of FAs on the surface roughness of the KCZ film. It can be seen in Figure 8 that KC film had lower Ra and Rq values than KGM film. This may be probably because CTS was relatively more compatible with KGM. Incorporating Zein led to an increase in the surface roughness of the KGM film. However, Ra and Rq values of KCZ film decreased when compared with KZ film, which may be due to the increasing number of hydrophilic groups on the surface. In addition, it was discovered that KCZ-FA films had higher Ra and Rq values than KCZ and KZ films, suggesting that the surface roughness of KCZ-FA films increased. Nevertheless, the Ra and Rq values of KCZ-FA films exhibited different variation patterns. The Ra and Rq values of KCZ-LA films increased with the increase in LA concentrations, whereas the Ra and Rq values of KCZ-SA films increased first and then decreased as the SA concentrations increased. It was also observed that the Ra and Rq values of KCZ-LA film were higher than those of KCZ-SA film when SA and LA concentrations were both 0.1%. The results indicated that the surface roughness of KCZ film was enhanced after adding FAs, as consistent with SEM results.

### 3.6. Mechanical Properties of Various Films

The effects of FAs on the mechanical properties of KCZ film are shown in Table 1. It was observed that adding CTS or Zein to pure KGM film caused an increase in the TS and EB values of binary composite films, whereas binary composite films had lower TS and higher EB values than KCZ film. In addition, it was found that the TS values of KCZ-FA films increased first and then decreased with the increase in FA concentrations, but only KCZ-LA5 film was discovered to have lower TS value than KCZ film. This is probably because a small amount of fatty acids could be evenly distributed in the film matrix [51], but the heterogeneity of excessive fatty acids weakened intermolecular interactions [47]. Nevertheless, the EB values of KCZ-FA films decreased first and then increased with the increase in FA concentrations. Only KCZ-LA2 and KCZ-LA3 films had lower EB values than KCZ film. It was also discovered that KCZ-FA films had the highest TS and lowest EB values when the concentrations of LA and SA were 0.3% and 0.06%, respectively. More importantly, KCZ-SA film was found to have higher TS and EB values than KCZ-LA film when SA and LA concentrations were both 0.1%. These results suggested that the mechanical properties of KCZ film could be improved after adding appropriate concentrations of FAs.

### 3.7. Water Content (WC), Solubility (WS), Vapor Permeability (WVP) and Contact Angle (WCA) of Various Films

The effects of FAs on the WVP, WC, and WS values of KCZ film are shown in Table 2. As seen from Table 2, the WVP value of pure KGM film was relatively large, suggesting that pure KGM film possessed poor water vapor barrier properties. However, the WVP values of KC, KZ, and KCZ films were significantly reduced (*p* < 0.05) when compared to pure KGM film. Moreover, it was found that the WVP values of KCZ-FA films decreased first and then increased with the increase in FA concentrations but were significantly lower (*p* < 0.05) than that of KCZ film, indicating that KCZ-FA films had better water vapor barrier properties than KCZ film. KCZ-LA and KCZ-SA films had the lowest WVP values when LA and SA concentrations were 0.3% and 0.06%, respectively. This is probably because the effective path length of water diffusion was increased due to the discontinuities in the hydrophilic phase by the dispersion of a small amount of fatty acids in the film matrix, resulting in the decrease in WVP values [52]. Nevertheless, the discontinuous crystallization and uneven emulsification of fatty acids appeared on the film surface when an excessive amount of fatty acids was added, thereby increasing the WVP values of the films [53,54]. More importantly, KCZ-SA and KCZ-LA films were discovered to have similar water vapor barrier properties when SA and LA concentrations were both 0.1%. From the above results, it could be concluded that the WVP values of KCZ film were not affected by FA types but were influenced by FA concentrations.

It was also observed from Table 2 that WC and WS values showed similar trends as the WVP values. The WC and WS values of pure KGM film were 53.89% and 100%, respectively, suggesting that pure KGM film had strong hydrophilicity due to a large number of hydroxyl groups in the chain of KGM molecules [12]. In addition, it was discovered that the WC and WS values of binary composite films were significantly lower (*p* < 0.05) than those of pure KGM film but were significantly higher (*p* < 0.05) than those of KCZ film. It was also found that the WC and WS values of KCZ-FA films decreased first and then increased with the increase in FA concentrations but were significantly lower (*p* < 0.05) than those of KCZ film. KCZ-LA and KCZ-SA films had the lowest WC and WS values when the concentrations of LA and SA were 0.3% and 0.06%, respectively. This may be probably because the KGM–CTS–Zein–water interactions were limited by the intermolecular interactions among KGM, CTS, Zein, and FAs after adding FAs, resulting in improving the hydrophobic properties of KCZ film. It could be concluded that KCZ-FA films had better hydrophobic and water vapor barrier properties.

Figure S1 shows the effects of FAs on the WCA values of KCZ film. As seen from Figure S1, the WCA values of KGM, KC, KZ, and KCZ films were all lower than 85°, indicating that they had poor hydrophobicities. It was also discovered that the WCA values of KCZ-FA films increased first and then decreased with the increase in FA concentrations but were significantly higher (*p* < 0.05) than that of KCZ film, indicating that KCZ-FA films had stronger hydrophobicities than KCZ film. KCZ-LA and KCZ-SA films had the highest WCA values when the concentrations of LA and SA were 0.3% and 0.06%, respectively. This is probably due to the formation of hydrogen bonds among KGM, CTS, Zein, and FAs. More importantly, KCZ-SA film was found to have a higher WCA value than KCZ-LA film when SA and LA concentrations were both 0.1%. These results indicated that the hydrophobicity of KCZ film was significantly influenced by FA types and concentrations.

### 3.8. Opacity and Color of Various Films

Table 3 shows the effects of FAs on the opacity of KCZ film. It could be seen from Table 3 that the opacities of binary composite films were higher than those of the corresponding pure films but were lower than that of KCZ film. This is probably because the strong interactions among KGM, CTS, and Zein made the film much denser, resulting in the decreasing transparency of KCZ film. In addition, it was found that KCZ-FA films exhibited higher opacities than KCZ film, and their opacities significantly (*p* < 0.05) increased with the increase in FA concentrations, indicating that KCZ-FA films had better light barrier properties than KCZ film. This may be probably because of poor light transmittance of fatty acids in a solid state at room temperature [51], light scattering generated by the dispersed fatty acids, and structural damage of the polymer chains to different degrees caused by different distributions of different fatty acids in the films [55,56]. More importantly, KCZ-SA film was discovered to have higher opacity than KCZ-LA film when SA and LA concentrations were both 0.1%. The results suggested that incorporating appropriate concentrations of SA could better decrease the transparency of KCZ film. This may be attributed to the fact that the alkyl chain of SA was longer than that of LA, resulting in poorer solubility and light transmittance.

The effects of FAs on the color of KCZ film are also presented in Table 3. It was observed that, except for the KCZ-LA1 film, all the other KCZ-FA films had lower L* and higher a* values than the KCZ film, which indicated the tendency of these KCZ-FA films toward dark redness. However, the b* and ΔE* values of KCZ-FA films were significantly higher than those of KCZ film (*p* < 0.05), suggesting that KCZ-FA films were more yellowed and colored. Moreover, the b* and ΔE* values of KCZ-FA films significantly (*p* < 0.05) increased with the increase in FA concentrations. The results indicated that the yellowness of KCZ-FA films increased with the increase in FA concentrations. It was also discovered that KCZ-SA film had higher b* and ΔE* values than KCZ-LA film when SA and LA concentrations were both 0.1%. The results showed that the color of KCZ-SA films was relatively much yellower than that of KCZ-LA films.

**Figure 9:**
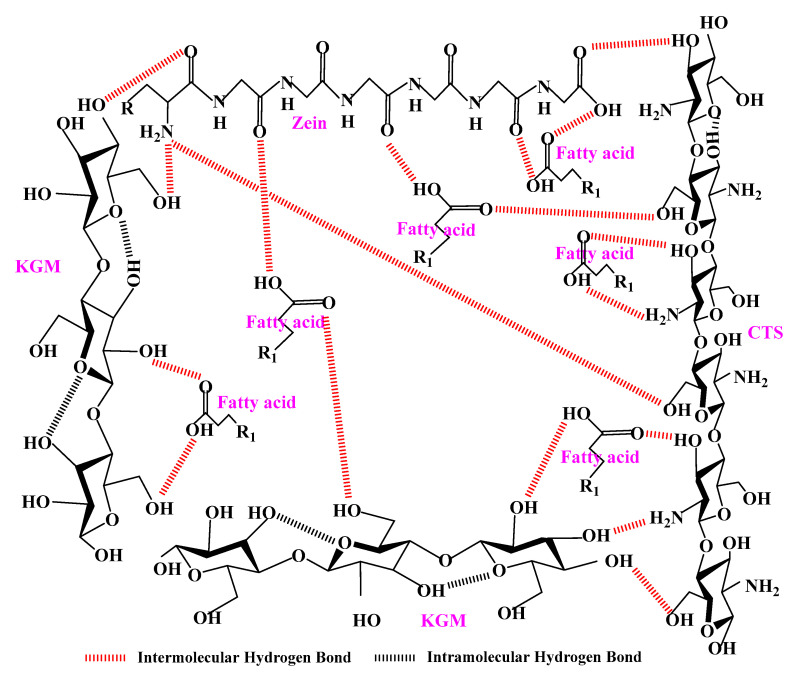
The potential formation mechanism of KCZ-FA films.

According to the above results, it could be concluded that the performances of KCZ film were effectively improved after incorporating fatty acids. Moreover, the potential formation mechanism of KCZ-FA films was presented in Figure 9. It was found that fatty acids could interact with KGM, Zein, and CTS, and the hydrogen bond interactions were formed among them. Therefore, this study contributes to a better understanding of KCZ-FA films with superior properties and provides a theoretical basis for the fabrication of food packaging films enriched with hydrophobic substances.

## 4. Conclusions

This paper is the first investigation of incorporating fatty acids into KGM/CTS/Zein film for better properties by a solution casting method. The addition of fatty acids improved the apparent viscosities of the film solution, thermal stabilities, crystallinities, color attributes, hydrophobicity, water vapor barrier, and mechanical properties of KCZ film. Incorporating fatty acids also significantly affected the surface morphologies and roughness of KCZ film. Hydrogen bond interactions among KGM, CTS, Zein, and FA molecules were confirmed by FTIR. Moreover, the opacities of KCZ-FA films were significantly higher than that of KCZ film and increased with the increase in FA concentrations. These results indicated that KCZ-FA films with enhanced properties exhibited great potential as biodegradable packaging materials for the preservation of various foods. However, this paper does not evaluate essential aspects for commercial applications, such as microbial resistance and the interaction of the films with different types of food. Therefore, further research will focus on studying the practical applications of KCZ-FA films in food packaging.

## Figures and Tables

**Table 1:** Mechanical properties of various films.

Films	Thickness (μm)	TS (MPa)	EB (%)
KGM	55.50 ± 5.26 ^b^	29.05 ± 0.31 ^f^	5.99 ± 0.23 ^ce^
CTS	71.50 ± 10.41 ^a^	17.00 ± 0.03 ^g^	3.64 ± 0.15 ^df^
Zein	45.00 ± 4.76 ^cde^	3.44 ± 0.13 ^h^	2.61 ± 0.30 ^df^
KC	46.75 ± 6.70 ^cde^	31.58 ± 0.27 ^d^	6.96 ± 0.37 ^ce^
KZ	52.75 ± 3.40 ^bc^	29.92 ± 0.16 ^e^	7.16 ± 0.40 ^ce^
KCZ	48.00 ± 5.42 ^bcd^	32.13 ± 0.02 ^cd^	6.34 ± 0.61 ^ce^
KCZ-LA1	49.50 ± 3.70 ^bcd^	32.76 ± 0.34 ^c^	8.71 ± 0.44 ^b^
KCZ-LA2	46.25 ± 6.85 ^cde^	34.49 ± 0.48 ^b^	5.91 ± 0.56 ^c^
KCZ-LA3	45.00 ± 4.76 ^cde^	37.94 ± 0.12 ^a^	2.82 ± 0.46 ^d^
KCZ-LA4	46.00 ± 5.89 ^cde^	34.46 ± 0.09 ^b^	7.17 ± 0.53 ^c^
KCZ-LA5	50.00 ± 1.63 ^bcd^	30.62 ± 0.35 ^e^	14.69 ± 0.39 ^a^
KCZ-SA2	48.25 ± 6.65 ^bcd^	33.10 ± 0.30 ^c^	16.15 ± 0.45 ^b^
KCZ-SA4	44.00 ± 6.06 ^de^	35.21 ± 0.45 ^b^	12.72 ± 0.28 ^c^
KCZ-SA6	38.25 ± 3.30 ^e^	37.59 ± 0.22 ^a^	9.34 ± 0.56 ^d^
KCZ-SA8	45.00 ± 7.02 ^cde^	34.66 ± 0.26 ^b^	10.08 ± 0.41 ^d^
KCZ-SA10	49.25 ± 9.78 ^bcd^	33.79 ± 0.17 ^c^	19.77 ± 0.37 ^a^

Values are the means ± standard deviations. The different letters in a column indicate significant differences at *p* < 0.05.

**Table 2:** Water content, solubility, and vapor permeability values of various films.

Films	WC (%)	WS (%)	WVP × 10^−11^ (g∙m^−1^·s^−1^·Pa^−1^)
KGM	53.89 ± 0.09 ^b^	100 ± 0.00 ^a^	9.31 ± 0.05 ^b^
CTS	68.30 ± 0.07 ^a^	46.35 ± 0.06 ^c^	9.97 ± 0.02 ^a^
Zein	9.50 ± 0.15 ^k^	10.61 ± 0.08 ^k^	6.25 ± 0.01 ^hi^
KC	53.20 ± 0.09 ^c^	47.63 ± 0.03 ^b^	9.25 ± 0.00 ^c^
KZ	42.45 ± 0.03 ^d^	38.77 ± 0.05 ^d^	8.82 ± 0.03 ^d^
KCZ	40.39 ± 0.03 ^e^	37.66 ± 0.04 ^e^	8.68 ± 0.05 ^e^
KCZ-LA1	36.73 ± 0.00 ^f^	34.31 ± 0.00 ^g^	7.60 ± 0.05 ^f^
KCZ-LA2	21.54 ± 0.02 ^i^	32.13 ± 0.13 ^i^	6.28 ± 0.01 ^h^
KCZ-LA3	16.11 ± 0.08 ^j^	30.79 ± 0.05 ^j^	4.58 ± 0.03 ^j^
KCZ-LA4	23.89 ± 0.06 ^h^	33.55 ± 0.07 ^h^	5.58 ± 0.02 ^i^
KCZ-LA5	35.78 ± 0.04 ^g^	36.15 ± 0.05 ^f^	6.86 ± 0.03 ^g^
KCZ-SA2	36.36 ± 0.00 ^f^	32.66 ± 0.12 ^g^	7.83 ± 0.02 ^f^
KCZ-SA4	35.34 ± 0.07 ^h^	31.33 ± 0.01 ^i^	7.02 ± 0.01 ^h^
KCZ-SA6	33.33 ± 0.00 ^j^	30.21 ± 0.09 ^j^	6.17 ± 0.01 ^i^
KCZ-SA8	34.44 ± 0.04 ^i^	32.31 ± 0.14 ^h^	7.32 ± 0.03 ^g^
KCZ-SA10	35.79 ± 0.15 ^g^	33.89 ± 0.05 ^f^	7.77 ± 0.02 ^f^

Values are the means ± standard deviations. The different letters in a column indicate significant differences at *p* < 0.05.

**Table 3:** Opacity and color of various films.

Samples	Opacity	L*	a*	b*	ΔE*
KGM	3.43 ± 0.03 ^jk^	92.59 ± 0.04 ^a^	0.82 ± 0.04 ^b^	−0.54 ± 0.03 ^k^	1.83 ± 0.04 ^k^
CTS	4.65 ± 0.05 ^ij^	92.46 ± 0.02 ^b^	0.42 ± 0.07 ^cd^	1.24 ± 0.02 ^j^	2.73 ± 0.02 ^j^
Zein	6.60 ± 0.08 ^hi^	90.50 ± 0.02 ^c^	0.79 ± 0.02 ^b^	5.75 ± 0.02 ^i^	6.58 ± 0.02 ^i^
KC	5.15 ± 0.08 ^gh^	92.36 ± 0.06 ^b^	0.49 ± 0.04 ^cd^	0.91 ± 0.08 ^h^	2.42 ± 0.11 ^h^
KZ	7.13 ± 0.03 ^fg^	90.11 ± 0.02 ^de^	0.97 ± 0.01 ^a^	6.07 ± 0.03 ^g^	6.93 ± 0.02 ^g^
KCZ	7.53 ± 0.07 ^ef^	90.38 ± 0.03 ^cd^	0.22 ± 0.01 ^ef^	7.08 ± 0.04 ^f^	7.96 ± 0.03 ^f^
KCZ-LA1	7.68 ± 0.01 ^e^	90.37 ± 0.03 ^c^	0.17 ± 0.05 ^f^	8.39 ± 0.01 ^e^	9.27 ± 0.01 ^e^
KCZ-LA2	8.00 ± 0.05 ^d^	89.81 ± 0.05 ^e^	0.32 ± 0.07 ^de^	9.57 ± 0.05 ^d^	10.46 ± 0.05 ^d^
KCZ-LA3	9.06 ± 0.13 ^c^	89.16 ± 0.04 ^f^	0.32 ± 0.04 ^de^	10.78 ± 0.03 ^c^	11.80 ± 0.03 ^c^
KCZ-LA4	11.19 ± 0.08 ^b^	88.70 ± 0.08 ^g^	0.36 ± 0.00 ^cd^	11.49 ± 0.05 ^b^	12.54 ± 0.02 ^b^
KCZ-LA5	13.19 ± 0.05 ^a^	88.41 ± 0.03 ^h^	0.45 ± 0.03 ^cd^	12.60 ± 0.00 ^a^	13.64 ± 0.00 ^a^
KCZ-SA2	11.22 ± 0.08 ^e^	88.67 ± 0.05 ^f^	0.39 ± 0.01 ^d^	9.69 ± 0.04 ^e^	10.60 ± 0.04 ^e^
KCZ-SA4	12.65 ± 0.02 ^d^	89.29 ± 0.02 ^g^	0.48 ± 0.05 ^cd^	10.59 ± 0.01 ^d^	11.52 ± 0.01 ^d^
KCZ-SA6	13.68 ± 0.06 ^c^	89.16 ± 0.00 ^h^	0.57 ± 0.09 ^c^	11.49 ± 0.01 ^c^	12.43 ± 0.00 ^c^
KCZ-SA8	14.28 ± 0.08 ^b^	88.83 ± 0.03 ^i^	0.58 ± 0.04 ^c^	12.35 ± 0.04 ^b^	13.32 ± 0.04 ^b^
KCZ-SA10	15.24 ± 0.04 ^a^	88.38 ± 0.06 ^j^	0.52 ± 0.03 ^cd^	13.58 ± 0.02 ^a^	14.66 ± 0.08 ^a^

Values are the means ± standard deviations. The different letters in a column indicate significant differences at *p* < 0.05.

## Data Availability

The original contributions presented in the study are included in the article, further inquiries can be directed to the corresponding author.

## Supplementary Materials

The following supporting information can be downloaded at: https://www.mdpi.com/article/10.3390/foods14091563/s1, Figure S1: Water contact angle of various films.

## Abbreviations

The following abbreviations are used in this manuscript:

KGM	Konjac glucomannan
CTS	Chitosan
SA	Stearic acid
LA	Lauric acid
FA	Fatty acid
KCZ	Konjac glucomannan/chitosan/Zein
WC	Water content
WS	Water solubility
WVP	Water vapor permeability
WCA	Water contact angle
EB	Elongation at break
TS	Tensile strength
FTIR	Fourier transform infrared spectroscopy
DSC	Differential scanning calorimetry
TGA	Thermogravimetric analyzer
XRD	X-ray diffractometer
SEM	Scanning electron microscopy
AFM	Atomic force microscope
