# Supplementary figures and images for: Incorporating Fatty Acids Enhanced the Performance of Konjac Glucomannan/Chitosan/Zein Film
Source: Foods. 2025 Apr 29;14(9):1563. doi: 10.3390/foods14091563 (PMC12072034; doi:10.3390/foods14091563)

## Supplementary Materials

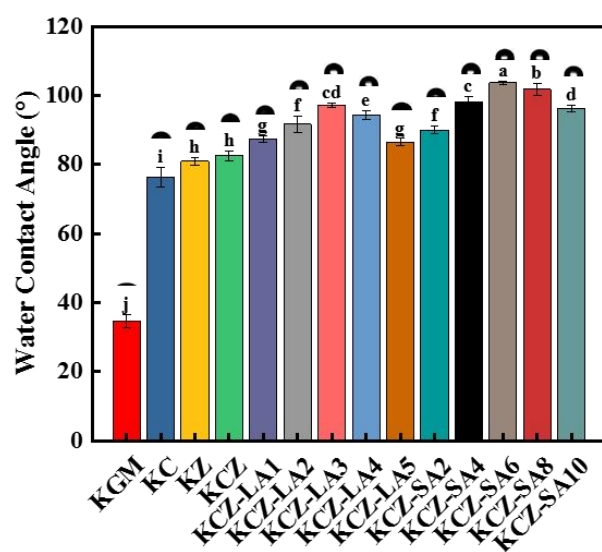

**Figure S1.** Water contact angle of various films.

Supplement: Supplementary file 1 [file foods-14-01563-s001.zip › foods-3544400-supplementary.pdf]
